# Association of *RAGE* gene multiple variants with the risk for COPD and asthma in northern Han Chinese

**DOI:** 10.18632/aging.101975

**Published:** 2019-05-29

**Authors:** Hongtao Niu, Wenquan Niu, Tao Yu, Feng Dong, Ke Huang, Ruirui Duan, Shiwei Qumu, Minya Lu, Yong Li, Ting Yang, Chen Wang

**Affiliations:** 1 Peking University China-Japan Friendship School of Clinical Medicine, Beijing 100029, China; 2 Department of Pulmonary and Critical Care Medicine, China-Japan Friendship Hospital, Beijing 100029, China; 3 Institute of Clinical Medical Sciences, China-Japan Friendship Hospital, Beijing 100029, China; 4 National Clinical Research Center for Respiratory Diseases, Beijing 100029, China; 5 Institute of Respiratory Medicine, Chinese Academy of Medical Sciences, Beijing 100029, China; 6 Clinical Diagnosis Department of Respiratory Diseases Center, China-Japan Friendship Hospital, Beijing 100029, China

**Keywords:** COPD, asthma, haplotype, variant, RAGE

## Abstract

Clinical and experimental data have shown that the receptor for advanced glycation end products (RAGE) is implicated in the pathogenesis of respiratory disorders. In this study, we genotyped five widely-evaluated variants in *RAGE* gene, aiming to assess their association with the risk for chronic obstructive pulmonary disease (COPD) and asthma in northern Han Chinese. Genotypes were determined in 105 COPD patients, 242 asthma patients and 527 controls. In single-locus analysis, there was significant difference in the genotype distributions of rs1800624 between COPD patients and controls (*p*=0.022), and the genotype and allele distributions of rs1800625 differed significantly (*p*=0.040 and 0.016) between asthma patients and controls. Haplotype analysis revealed that haplotype T-A-G-T (allele order: rs1800625, rs1800624, rs2070600, rs184003) was significantly associated with a reduced COPD risk (OR=0.32, 95% CI: 0.06-0.60), and haplotype T-A-A-G was significantly associated with a reduced asthma risk (OR=0.19, 95% CI: 0.04-0.96). Further haplotype-phenotype analysis showed that high- and low-density lipoprotein cholesterol and blood urea nitrogen were significant mediators for COPD (*p*_sim_=0.041, 0.043 and 0.030, respectively), and total cholesterol was a significant mediator for asthma (*p*_sim_=0.009). Taken together, our findings indicate that *RAGE* gene is a promising candidate for COPD and asthma, and importantly both disorders are genetically heterogeneous.

## Introduction

Chronic obstructive pulmonary disease (COPD) and asthma are the two most common respiratory disorders, and they constitute chronic non-specific lung diseases (CNSLD) [1]. COPD and asthma share many phenotype similarities, such as airflow limitation, breathlessness, dyspnea, coughing, wheezing and chronic inflammation [2,3]. Evidence is mounting suggesting that COPD and asthma are complex multifactorial diseases, involving many environmental and genetic components [4,5]. Although many studies have evaluated the genetic underpinnings of COPD and asthma [6–10], it still remains a challengeable task to determine how many genes and which genetic alterations are actually involved in the pathogenesis of both disorders. An effective strategy is to identify disease-susceptibility genes that involve specific physiological or cellular function.

Recently, several genes have been documented to be simultaneously associated with the risk for COPD and asthma [11–13], indicating that COPD and asthma might share a common genetic origin that is possibly involved in the development of the lungs [14–17]. Experimental studies supported the strategies of inhibiting the receptor for advanced glycation end products (RAGE) in lung injury [18], and the contribution of RAGE to chronic inflammation, suggesting that RAGE may be a therapeutic target for lung-related diseases [19]. In transgenic mouse models, RAGE was identified to play a role in alveolar morphogenesis during lung development, and RAGE overexpression can cause the development of an emphysema-like phenotype in adult mice [20]. Epidemiological studies indicated that *RAGE* genetic variation was associated with the risk for COPD and asthma [21–24]. As the genomic sequences of *RAGE* gene are highly polymorphic, it is of added interest to determine which genetic variation in *RAGE* gene might have a functional role in regulating the bioavailability of RAGE, and thus the development of CNSLD. Importantly, two genome-wide association studies in healthy individuals of European ancestry reported a significant association between *RAGE* gene rs2070600 and spirometry measures of airflow obstruction [25,26]. By contrast, this variant was not significantly associated with asthma risk in another genome-wide association study in Japanese [27]. This discrepancy might reflect differences in genetic backgrounds across ethnic groups or in sampling strategies. Based on above evidence, we developed a hypothesis that *RAGE* gene may be a promising candidate in susceptibility to both COPD and asthma.

To test this hypothesis, we genotyped five widely-evaluated variants in *RAGE* gene, aiming to assess the association of these variants with the risk for COPD and asthma in a population-based cohort from northern China.

## RESULTS

### Baseline characteristics

The characteristics of study participants are shown in Table 1. No statistical difference was observed for the distributions of age and gender between patients and controls (both *p* >0.05). In contrast, COPD/asthma patients had significantly higher levels of body mass index, blood urea nitrogen and creatinine, yet significantly lower levels of plasma high-density lipoprotein cholesterol, homocysteine and uric acid than controls (all *p* <0.05). Higher levels of plasma low-density lipoprotein cholesterol, fasting plasma glucose and uric acid (all *p* <0.05) were found in COPD patients than controls. As expected, two key spirometry indexes, forced expiratory volume in 1 second (FEV_1_) (% of predicted) and FEV_1_/forced vital capacity (FVC), were significantly lower in patients diagnosed with COPD or asthma than in controls (both *p* <0.001).

**Table 1:** Baseline characteristics of study population.

**Characteristics**	**Controls**		**COPD + Asthma**			**COPD**			**Asthma**	
	**n=527**		**n=347**	p*		**n=105**	p*		**n=242**	p*
Age (years)	51.69±6.05		51.91±10.70^a^	0.690		57.73±7.77	0.110		49.39±10.85^a^	0.100
Gender (male, %)	247 (46.87)		142 (40.92)^b^	0.095		58 (55.24)	0.072		84 (34.71)^b^	0.109
BMI (kg/m^2^)	24.49 [22.18-26.53]		25.39 [23.31-28.30]	0.003		25.97 [23.80-27.66]	0.019		25.39 [23.15-28.63]	<0.001
FEV_1_/ FVC	81.40 [77.69-84.56]		68 [57-76.72]	<0.001		54.86 [46.44-64.08]	<0.001		74.00 [70.05-79.00]	<0.001
FEV1% pred	96.80 [92-100.7]		71.40 [51.20-89]	<0.001		49.79 [34–60]	<0.001		82.50 [67.30-97.50]	<0.001
TC (mmol/L)	4.80 [4.31-5.36]		4.85 [4.18-5.72]	0.392		4.91 [4.30-5.75]	0.124		4.82 [4.11-5.71]	0.856
TG (mmol/L)	1.38 [0.91-1.92]		1.32 [0.93-2.03]	0.982		1.46 [1.05-2.24]	0.180		1.29 [0.89-2]	0.465
HDLC (mmol/L)	1.33 [1.1-1.56]		1.14 [0.98-1.35]	<0.001		1.13 [1.01-1.28]	<0.001		1.16 [0.95-1.37]	<0.001
LDLC (mmol/L)	2.81 [2.44-3.34]		3.01 [2.42-3.65]	0.030		3.12 [2.64-3.65]	0.003		2.91 [2.36-3.62]	0.299
HCY (umol/L)	6.69 [5.80-7.80]		12.46 [10.51-14.86]	<0.001		13.42 [11.33-16.70]	<0.001		12.06 [10.24-14.57]	<0.001
FPG (mmol/L)	5.35 [5-5.81]		5.31 [4.79-6.19]	0.777		5.78 [4.97-6.69]	0.013		5.24 [4.79-5.95]	0.068
BUN (mmol/L)	5.75 [4.86-6.81]		4.69 [3.90-5.57]	<0.001		5.02 [4.26-5.65]	<0.001		4.55 [3.81-5.51]	<0.001
Creatinine (μmol/L)	66.35 [58.20-77.20]		60.35 [52.75-72.20]	<0.001		63.20 [54.90-74.30]	0.017		59.20 [51.20-71.09]	<0.001
Uric acid (μmol/L)	302 [250-361.5]		297 [246-368]	0.849		317.00 [266-394]	0.023		290 [238-344]	0.112

*p values were calculated using unpaired t-test. Data are presented as median [interquartile range] or mean ± SD, unless otherwise stated. Abbreviations: COPD, chronic obstructive pulmonary disease; BMI, Body mass index; FEV_1_, forced expiratory volume in 1 second; % pred, % predicted; TC, total cholesterol; TG, triglycerides; HDLC, high-density lipoprotein cholesterol; LDLC, low-density lipoprotein cholesterol; HCY, homocysteine; FPG, fasting plasma glucose; BUN, blood urea nitrogen.

### Single-locus analysis

Genotype frequencies of five studied variants in *RAGE* gene - rs1800625, rs1800624, rs2070600, rs184003 and rs2071288, satisfied the Hardy-Weinberg equilibrium in both patients and controls (all *p* >0.05). The pairwise linkage disequilibrium between five studied variants in all study participants, expressed as D’ and r^2^, is presented in Supplementary Figure 1. These variants were weakly linked (r^2^ <0.03).

The genotype and allele distributions of five studied variants in *RAGE* gene between asthma/COPD/both patients and controls are depicted in Table 2. For the comparison between COPD/asthma patients and controls, significance was only detected for the genotypes of rs1800624 (*p* =0.011). For the comparison between asthma patients and controls, there was significant difference in the genotype distributions of rs1800624 (*p* =0.022). For the comparison between COPD patients and controls, the genotype and allele distributions of rs1800625 differed significantly (*p* =0.040 and 0.016, respectively).

**Table 2:** The genotype/allele distributions of five studied variants in *RAGE* gene between patients and healthy controls.

**Variants**	**Genotype/allele**	**Controls**	**COPD + Asthma**	**χ^2^**	p*		**COPD**	**χ^2^**	p*		**Asthma**	**χ^2^**	p*
rs1800625	TT	379	262				88				182		
	CT	133	76	1.39	0.499		15	6.45	0.040		53	1.01	0.603
	CC	15	9				2				7		
	C (%)	15.47	13.55	1.23	0.267		9.05	5.85	0.016		13.84	0.69	0.408
rs1800624	TT	377	257				80				177		
	AT	131	88	8.19	0.011		24	2.35	0.353		64	6.72	0.022
	AA	19	2				1				1		
	A (%)	16.03	13.26	2.54	0.111		12.38	1.79	0.181		13.64	1.47	0.225
rs2070600	GG	359	233				72				161		
	AG	147	102	0.35	0.840		30	0.31	0.939		72	0.29	0.864
	AA	21	12				3				9		
	A (%)	17.93	18.16	0.01	0.905		17.14	0.07	0.785		18.59	0.10	0.754
rs184003	GG	372	255				74				181		
	GT	142	86	1.14	0.565		27	0.64	0.684		59	3.11	0.229
	TT	13	6				4				2		
	T (%)	15.94	14.12	1.07	0.300		16.67	0.07	0.785		13.02	1.47	0.225
rs2071288	GG	510	339				102				237		
	AG	17	8	0.64	0.535		3	0.04	1.000		5	0.80	0.487
	AA	0	0				0				0		
	A (%)	1.61	1.15	0.63	0.428		1.42	0.04	1.000		1.03	0.79	0.374

*p values were calculated using χ^2^ test from a series of 3*2 contingency tables for genotype data and 2*2 contingency tables for allele data.

Abbreviations: COPD, chronic obstructive pulmonary disease; RAGE, the receptor for advanced glycation end products.

### Haplotype-disease analysis

Because of the low occurrence of rs2071288 mutant A allele in both patients and controls (Table 2), this variant was not included in further haplotype-disease and haplotype-phenotype analyses. As shown in Table 3, before and after adjusting for covariates including age, gender, body mass index, total cholesterol, triglyceride, high-density lipoprotein cholesterol, homocysteine and fasting plasma glucose, haplotype analysis revealed that the frequency of haplotype T-T-G-G (alleles in order of rs1800625, rs1800624, rs2070600 and rs184003, similarly hereinafter) was significantly higher in COPD/asthma patients than in controls (*p*_adj_. =0.032), while the frequencies of haplotypes T-A-A-G (*p*_adj_. =0.030) and T-A-G-T (*p*_adj_. =0.001) were significantly lower. Haplotype T-A-G-T was underrepresented in COPD patients relative to in controls (*p*_adj_. =0.013), and by contrast haplotype T-A-A-G was underrepresented in asthma patients relative to controls (*p*_adj_. =0.004). There was no detectable significance for the other haplotypes between patients and controls.

**Table 3:** Distributions of estimated haplotypes (frequency >1%) of four studied variants in *RAGE* gene between patients and healthy controls.

**Haplotype^a^** **(%)**	**Controls**	**COPD and Asthma**					**COPD**					**Asthma**			
		**Patients**	**Hap. score**	***p***	***p*_adj_.^b^**		**Patients**	**Hap. score**	***p***	***p*_adj_.^b^**		**Patients**	**Hap. score**	***p***	***p*_adj_. ^b^**
T-T-G-G	41.41	47.04	2.15	0.032	0.032		46.90	2.07	0.039	0.054		45.40	2.03	0.042	0.986
T-T-A-G	14.38	16.42	0.98	0.329	0.329		15.70	0.91	0.361	0.333		15.80	-0.05	0.964	0.492
T-T-G-T	13.28	11.67	-0.73	0.466	0.325		12.30	-0.78	0.437	0.428		13.70	0.34	0.732	0.667
C-T-G-G	12.42	9.45	-1.12	0.263	0.462		9.70	-1.10	0.269	0.263		10.70	-1.02	0.306	0.145
T-A-G-G	7.69	8.16	-0.64	0.525	0.787		8.60	-0.14	0.888	0.983		7.80	-0.44	0.662	0.346
T-A-A-G	3.88	0.83	-2.70	0.007	0.030		2.00	-1.67	0.095	0.122		1.00	-2.62	0.009	0.004
T-A-G-T	2.49	0.34	-2.32	0.021	0.001		0.00	-2.48	0.013	0.013		0.40	-1.54	0.124	0.081
C-A-G-G	2.43	2.74	-0.32	0.750	0.929		2.60	-0.85	0.395	0.426		1.80	-0.65	0.514	0.321

^a^Alleles in each haplotype were assigned in order of rs1800625, rs1800624, rs2070600 and rs184003.

^b^Adjusted *p* values (*p*_adj_.) for age, gender, body mass index, total cholesterol; high-density lipoprotein cholesterol and fasting plasma glucose.

Abbreviations: COPD, chronic obstructive pulmonary disease; RAGE, the receptor for advanced glycation end products.

Additionally, we calculated the prediction of above haplotypes for the risk of COPD and asthma and both (Table 4). Taking haplotype T-T-G-G as a reference, haplotypes T-A-G-G (odds ratio [OR]=0.19, 95% confidence interval [CI]: 0.04-0.94) and T-A-G-T (OR=0.16, 95% CI: 0.01-2.96) were associated with a significantly lower risk of COPD or asthma. For COPD, haplotype T-A-G-T was significantly associated with a reduced risk (OR=0.32, 95% CI: 0.06-0.60). For asthma, haplotype T-A-A-G was significantly associated with a reduced risk (OR=0.19, 95% CI: 0.04-0.96).

**Table 4:** Prediction of estimated haplotypes (frequency >1%) of four studied variants in *RAGE* gene for the risk of asthma and COPD.

**Haplotype^a^**	**COPD + Asthma**	**COPD**	**Asthma**
T-T-G-G	Reference haplotype		
T-T-A-G	1.08 (0.79-1.49) 0.329	1.07 (0.78-1.46) 0.333	0.94 (0.62-1.43) 0.492
T-T-G-T	0.83 (0.57-1.20) 0.325	0.93 (0.66-1.29) 0.428	0.93 (0.58-1.49) 0.667
C-T-G-G	0.66 (0.44-0.97) 0.462	0.72 (0.50-1.05) 0.263	0.66 (0.40-1.11) 0.145
T-A-G-G	0.19 (0.04-0.94) 0.030	0.97 (0.63-1.48) 0.983	0.86 (0.44-1.69) 0.346
T-A-A-G	0.87 (0.55-1.36) 0.787	0.50 (0.21-1.19) 0.122	0.19 (0.04-0.96) 0.004
T-A-G-T	0.16 (0.01-2.96) 0.001	0.32 (0.06-0.60) 0.013	0.34 (0.05-2.53) 0.081
C-A-G-G	1.23 (0.58-2.62) 0.929	1.01 (0.49-2.08) 0.426	0.90 (0.32-2.54) 0.321

^a^Alleles in each haplotype were assigned in order of rs1800625, rs1800624, rs2070600 and rs184003.

Data are expressed as odds ratio (95% confidence interval) *p* value. Abbreviations: COPD, chronic obstructive pulmonary disease; RAGE, the receptor for advanced glycation end products.

### Haplotype-phenotype analysis

When considering above haplotypes of four variants in *RAGE* gene as a whole, the omnibus tests for the association between haplotypes and all baseline characteristics before and after simulation correction are presented in Table 5. In COPD/asthma patients, significant association was found for total cholesterol (*p*_sim_ = 0.008). In COPD patients, association was significant for high-density lipoprotein cholesterol, low-density lipoprotein cholesterol and blood urea nitrogen (*p*_sim_ =0.041, 0.043 and 0.030, respectively). In asthma patients, only total cholesterol was significantly associated with all haplotypes (*p*_sim_ =0.009).

**Table 5:** Global testing of haplotypes of four studied variants in *RAGE* gene as a whole with anthropometric indexes and clinical biomarkers in COPD and asthma patients.

**Variables**	**COPD + Asthma**				**COPD**				**Asthma**		
	**Global statistics**	***p***	***p*_sim_**		**Global statistics**	***p***	***p*_sim_**		**Global statistics**	***p***	***p*_sim_**
Age (years)	20.45	0.117	0.160		15.16	0.298	0.234		20.45	0.117	0.120
Gender (male)	11.36	0.658	0.687		21.29	0.067	0.066		11.36	0.658	0.681
BMI (kg/m^2^)	8.47	0.864	0.683		4.03	0.983	0.884		8.47	0.864	0.670
TC (mmol/L)	65.59	<0.001	0.008		22.70	0.454	0.095		65.59	<0.001	0.009
TG (mmol/L)	12.29	0.583	0.288		7.63	0.867	0.685		12.29	0.583	0.329
HDLC (mmol/L)	13.19	0.512	0.420		24.55	0.026	0.041		13.19	0.512	0.427
LDLC (mmol/L)	0.59	1.000	0.954		32.34	0.002	0.043		0.59	1.000	0.949
HCY (umol/L)	13.83	0.463	0.241		15.58	0.272	0.159		13.83	0.463	0.258
FPG (mmol/L)	4.18	0.997	0.821		7.46	0.877	0.570		4.18	0.997	0.837
BUN (mmol/L)	11.72	0.629	0.548		30.90	0.004	0.030		11.72	0.629	0.556
Creatinine (μmol/L)	2.66	0.999	0.922		7.42	0.879	0.795		2.66	0.999	0.927
Uric acid (μmol/L)	8.11	0.884	0.777		4.89	0.978	0.866		8.11	0.884	0.774

Abbreviations: COPD, chronic obstructive pulmonary disease; RAGE, the receptor for advanced glycation end products; BMI, body mass index; TC, total cholesterol; TG, triglycerides; HDLC, high-density lipoprotein cholesterol; LDLC, low-density lipoprotein cholesterol; HCY, homocysteine; FPG, fasting plasma glucose; BUN, blood urea nitrogen.

### Nomogram presentation

**Figure 1:**
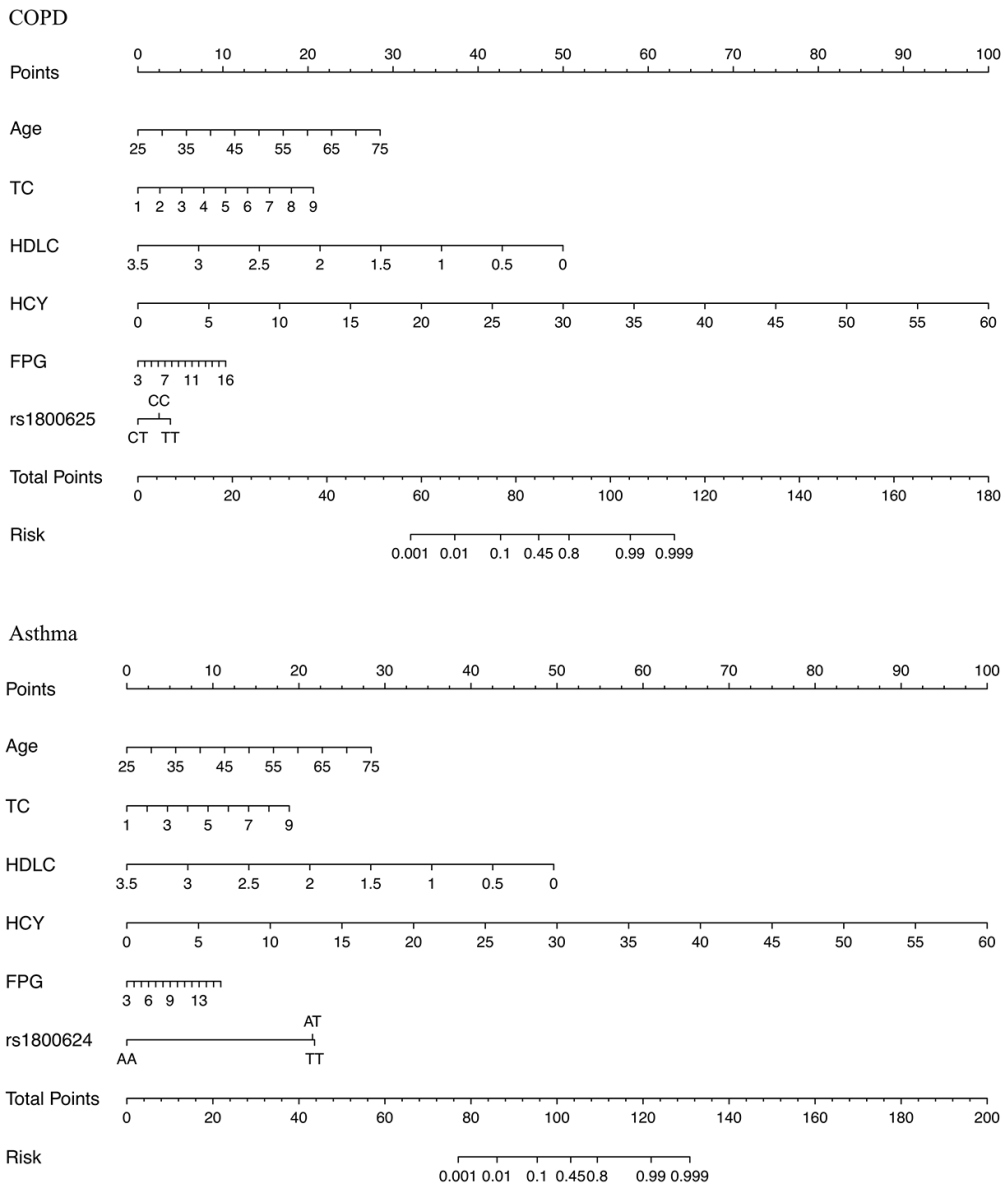
The nomogram graphs for estimating the risk of COPD (the upper panel) and asthma (the lower panel) based on significant risk factors. Abbreviations: COPD: chronic obstructive pulmonary disease; TC: total cholesterol; HDLC: high-density lipoprotein cholesterol; HCY: homocysteine; FPG: fasting plasma glucose. The point for each factor is summed and projected on total point line. A vertical line is projected from total point line to predicted probability bottom scale to obtain the individual probability of COPD or asthma risk.

The prediction nomogram graphs that integrated all significant risk factors of COPD and asthma are illustrated in Figure 1. The risk factors included age of onset, total cholesterol, high-density lipoprotein cholesterol, homocysteine, fasting plasma glucose and rs1800625 (for COPD only) and rs1800624 (for asthma only). Specifically, a point was assigned for each risk factor, and a total point, calculated from the sum of individual points, was visually indicated as a predictive probability for COPD or asthma.

## DISCUSSION

The aim of this study was to assess the association of *RAGE* genetic variation with the risk for COPD and asthma in a population-based cohort from northern China. Our findings supported the hypothesis that *RAGE* gene is a promising candidate for COPD and asthma. It is worth noting that the risk profile in *RAGE* gene differs between COPD and asthma, as our single-locus analysis revealed a significant association of rs1800625 with COPD and rs1800624 with asthma, indicating that COPD and asthma are genetically heterogeneous respiratory disorders, the findings being further reinforced by our haplotype-disease and haplotype-phenotype analyses.

In humans, the gene encoding RAGE (Gene ID: 177) is located on short arm of chromosome 6 (6p21.3), and it spans 3.27 kb comprising 11 exons [28]. Since its discovery in 1992 [29], RAGE has been extensively assessed in susceptibility to various disease conditions [22,30]. RAGE is a 35 kilodalton transmembrane receptor of the immunoglobulin superfamily, and it is widely expressed, predominantly in the lungs [31,32]. Experimental data indicated that RAGE is mainly presented on vascular smooth muscle cells [33], airway smooth muscle cells (ASM), endothelial cells and pulmonary macrophages [34]. Additionally, there is clinical evidence that neutrophilic airway inflammation in COPD and asthma is associated with reduced soluble RAGE [35]. It is hence reasonable to speculate that RAGE is implicated in the pathogenesis of respiratory disorders.

In the medical literature, a growing number of studies have shown that RAGE plays a critical role in physiological and pathological processes of the lungs, and variants in *RAGE* gene may predispose to the risk of respiratory disorders, including COPD and asthma [36–39]. However, the results of most published studies remain inconsistent and inconclusive, with no consensus on genetic implications of *RAGE* gene, likely due to ethnic diversity of genetic backgrounds, lack of adjustment for confounders and disregard of haplotype analyses. The complex nature of COPD and asthma phenotypes requires additional validation in independent groups to establish the role of *RAGE* gene in the pathogenesis of both disorders, as well as to explore the effect of confounding factors.

The key findings of this association study identified two unlinked promoter variants in *RAGE* gene that were separately associated with the risk for COPD (rs1800625, -429T>C) and asthma (rs1800624, -374T>A) in northern Han Chinese. In contrast to a previous study by Li et al in southern Chinese, rs1800625 was not associated with the significant risk for COPD, and instead an exonic variant (rs2070600, G82S) was found to be a significant risk locus for COPD [22]. In addition, Guo et al observed that *RAGE* gene rs2070600 was associated with COPD risk in Shanghainese [40]. The possible reasons for observed contradictions might be due to the climate and cultural differences between northern and southern Chinese and patient selection. In this study, all study patients with either COPD or asthma were enrolled from natural populations, less susceptible to selection bias and population stratification. Compared with COPD, less is known regarding *RAGE* gene in susceptibility to asthma, except for several genome-wide association studies that did not support the contribution of *RAGE* gene to asthma risk [27,41]. Although some experimental and clinical studies have evaluated the pathophysiological role of RAGE in asthma [42–44], this study, to the best of our knowledge, is the first that has evaluated the association of *RAGE* genetic variants with asthma risk, and our findings identified a promoter marker in significant predisposition to asthma, which requires additional validation in other independent populations.

Besides single-locus analysis, we explored the haplotype-based association of four common variants in *RAGE* gene with the risk for COPD and asthma. In theory, haplotype analysis focuses on single genetic variants in their combination simultaneously and provides more information than single-locus analysis. Using haplotype technique, we identified two significant haplotypes that were differentially associated with the risk of both respiratory disorders, which reinforced the results of our single-locus analysis. What’s more, we investigated the association between derived haplotypes based on four common variants in *RAGE* gene and baseline characteristics, and we observed that high- and low-density lipoprotein cholesterol and blood urea nitrogen might mediate the association between haplotypes and COPD risk, and total cholesterol might be a mediator for asthma. Our observations are biologically plausible, as there is evidence that RAGE may contribute to the regulation of cholesterol homeostasis in macrophages and the involvement in hypercholesterolemia [45]. Moreover, elevated cholesterol levels were found to be association with an increased risk of COPD [46] and asthma [47]. Both haplotype-disease and haplotype-phenotype analyses support the notion that COPD and asthma are two heterogeneous respiratory disorders with different genetic profiles.

Some possible limitations should be acknowledged for this association study. First, the cross-sectional nature of this case-control association study precludes comments on causality. Second, we genotyped only five variants in *RAGE* gene, which might under-evaluate the contribution of this gene to the pathogenesis of COPD and asthma. Third, data on plasma soluble RAGE were unavailable for us to further interrogate its association with *RAGE* genotypes and haplotypes. Fourth, all participants enrolled in this study are currently living in the Beijing-Tianjin-Hebei region, where air pollution is a serious problem. Yet, we had no data on ambient air pollutants, which are established as significant risk factors for both COPD [48] and asthma [49]. Fifth, the sample size was not sufficiently enough to derive a reliable estimate, calling for further external validation. Sixth, our sample comprised exclusively northern Han Chinese, and hence our findings cannot be generalized to other ethnic groups.

Taken together, our findings indicate that *RAGE* gene is a promising candidate for COPD and asthma, and importantly the risk profiles in *RAGE* gene differ between COPD and asthma, indicating that both disorders are genetically heterogeneous. We hope that this study will not remain just another endpoint of scientific investigations instead of a start to establish background data for future studies on the association of *RAGE* genetic variants with COPD and asthma, the molecular mechanisms of RAGE in respiratory disorders.

## MATERIALS AND METHODS

### Study participants

**Figure 2:**
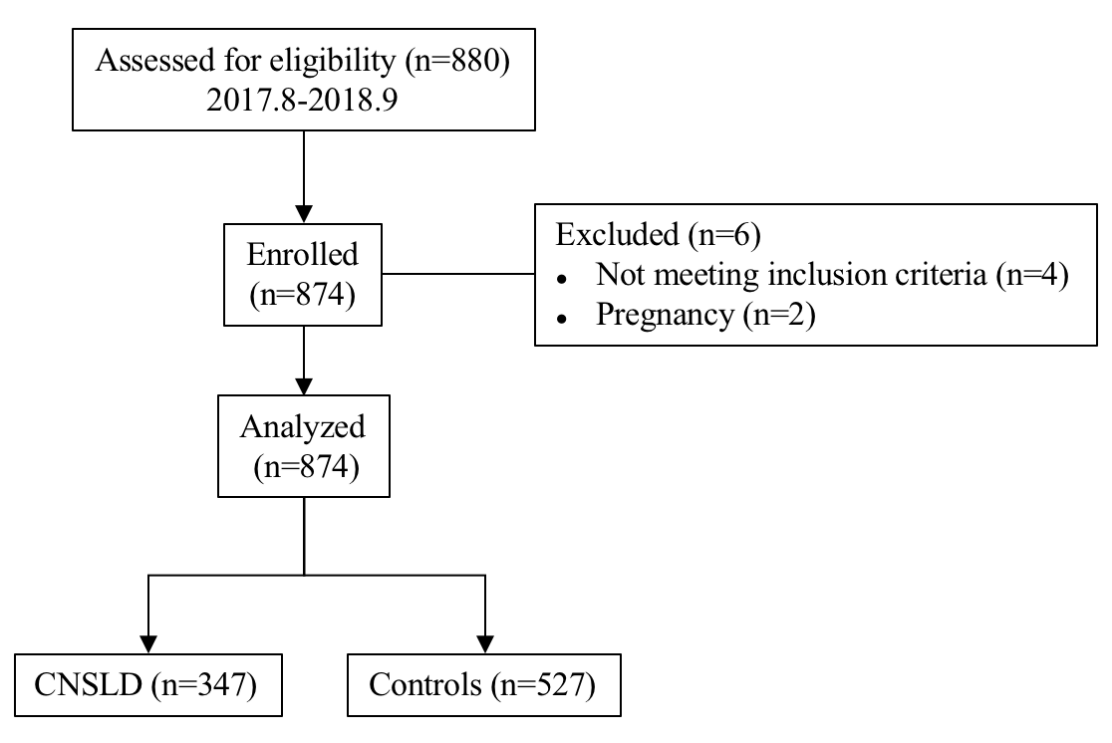
**Flow chart for the selection of participants in this case-control association study.** Abbreviations: CNSLD: chronic non-specific lung diseases.

This is a case-control genetic association study involving 105 patients with COPD and 242 patients with asthma, who participated in a multicenter research project. The control group was composed of 527 healthy individuals, without clinical evidence of COPD and asthma. All participants were self-reported as Han Chinese. COPD/asthma patients were frequency matched to controls on gender and age by random sampling. A detailed clinical history was recorded by using predesigned questionnaire. The selection process of all study participants is presented in Figure 2.

The study protocol was reviewed and approved by the Ethics Committee of China-Japan Friendship Hospital, and informed written consent was obtained from all participants.

### Diagnostic criteria

All study subjects were screened from August 2017 to September 2018 for eligibility in this study. COPD is diagnosed according to the Global Initiative for Chronic Obstructive Lung Disease (GOLD) [50], and asthma is diagnosed according to the Global Initiative for asthma (GINA) [51]. The diagnoses of COPD and asthma were made by using standard clinical criteria, and were further confirmed by spirometry, chest X-ray and Computed Tomography when necessary.

### Inclusion and exclusion criteria

Specifically, a patient was diagnosed to have COPD if he or she had a ratio of forced expiratory volume in the first second (FEV_1_) to forced vital capacity (FVC) of less than 0.7, which was measured 20 minutes after the administration of salbutamol. For asthma patients, diagnosis was made according to (a) medical reports of treating physicians; (b) symptoms; (c) use of medications for asthma; (d) reversible airflow limitation, and (e) FEV_1_ reversibility >12% and 200 ml after a post bronchodilator spirometry.

Healthy controls were enrolled from the same communities or local areas where patients lived. Controls were included if they had (a) no previous or present diagnosis of COPD, asthma and other respiratory diseases, (b) no history of wheezing, shortness of breath and other symptoms of allergic diseases including nasal and skin symptoms, and (c) no use of medications for COPD and asthma. Spirometry without bronchodilator was performed for controls.

Patients and controls were excluded if they (a) were diagnosed with cancer within the last 5 years, (b) had previous or actual episodes of venous thromboembolism, (c) received immobilization for more than 3 days, (d) were current or former smokers with an abstinence time less than 6 months, (e) had suspected acute inflammatory or infectious disease, (f) received anticoagulant therapy, (g) had diabetes mellitus, heart failure, chronic renal failure, liver disease, pregnancy, hormone replacement therapy, stroke and acute coronary syndrome.

### Demographic and clinical measurements

Body weight and height were measured with participants wearing minimal indoor clothing and bare feet. Body height was measured to the nearest 0.5 cm using a portable stadiometer. Body weight was measured to the nearest 0.1 kg using a standard scale. Body mass index was calculated as weight in kilograms divided by body height in meter squared.

Fasting venous blood was drawn to assay plasma glucose, plasma homocysteine, plasma triglyceride, total, high- and low-density lipoprotein cholesterol, blood urea nitrogen, creatinine and uric acid at the Laboratory Department of China-Japan Friendship Hospital (Beijing, China).

### Genotyping

Five candidate variants in *RAGE* gene, including rs1800625, rs1800624, rs2070600, rs184003 and rs2071288 were selected on the basis of their biological function [52,53]. In addition, these variants were widely evaluated in association with a wide range of clinical endpoints [28].

Magnetic bead technology was employed to extract genomic DNA by King Fisher Flex Purification System (Thermo Scientific, Waltham, MA) on robotic pipetting workstation (Tecan, Morrisville, NC). DNA was extracted from 874 samples of 200 μl of EDTA-treated whole blood using magnetic beads within 2 h. Working with magnetic particles can be divided into five separate processes: collecting magnetic particles, releasing magnetic particles, washing magnetic particles, incubation and concentration.

DNA quantity and quality were assessed by NanoDrop ND-1000 spectrophotometer (NanoDrop Technologies Inc., Wilmington, Delaware, USA). Absorbance was measured at wavelengths of 260 and 280 (A260 and A280, respectively) nm. The absorbance quotient (OD260/OD280) provides an estimate of DNA purity. An absorbance quotient value of 1.8 < ratio (R) < 2.0 was considered to be pure and high-quality DNA. A ratio below 1.8 is indicative of protein contamination, where as a ratio above 2.0 indicates ribonucleic acid (RNA) contamination. DNA samples were stored at -80°C in BioBank Center of China-Japan Friendship Hospital until mass assay.

PCR amplification was performed on A300 Peltier Thermal Cycler (LongGene Scientific Instruments Co., Ltd) containing 10 pmol of each primer. The forward and reverse primers for each SNP were shown in Supplementary Table 1. Primers were designed by Shanghai Generay Biotech Co.,Ltd (Shanghai, China). All PCR procedures were carried out under the following cycling conditions: initial denaturation at 94°C for 3 min, then 37 cycles of 94°C for 30 sec, 56°C for 30 sec and 72°C for 90 sec, followed by a final extension at 72°C for 5 min.

Variant detection was based on LDR (Ligase Detection Reaction) techniques. Two oligo DNA probes were connected only under the circumstance that the two probes are complementary to the target DNA sequences with the Taq DNA ligase and there was no any gap exist between the two probes, or the ligation reaction could not occur. SNP sites can be detected by scanning the length of product fragment by fluorescent.

### Statistical analysis

For database management, statistical calculation, and analysis, we used Stata software version 14.0 (StataCorp, TX, USA). Continuous variables were compared by the Student’s t-test or Wilcoxon test. Pearson (or Spearman when indicated) correlation was performed to evaluate potential relationship. The χ^2^ test was used to assess the goodness of fit between observed allele frequencies and expected counterparts by Hardy-Weinberg equilibrium, and to evaluate the differences in genotype/allele distributions between patients and controls.

Continuous data are expressed as mean ± standard deviation, or median with interquartile range, and a two-tailed *p* value less than 0.05 was considered significant. Odds ratio (OR) with 95% confidence interval (95% CI) was calculated for risk prediction, and Forward logistic regression analysis was performed to identify significant risk factors.

The extent of pairwise linkage disequilibrium between variants was calculated as D’ and r^2^ statistics using the Haploview software (version 4.0) (Cambridge, MA, USA).

A haplotype is defined as the combination of alleles for different variants that occur on the same chromosome. Haplo.em program was used to derive haplotype frequencies. Haplo.glm was employed to calculate OR and 95% CI for each haplotype. Haplo.score was used to model an individual's phenotype as a function of each inferred haplotype to account for haplotype ambiguity. Haplo.em, haplo.glm and haplo.score were completed by the Haplo.Stats software (v.1.4.0) using the R language (http://www.r-project.org) (version 3.5.2).

A nomogram was constructed on the basis of significant risk factors selected by Forward logistic regression analysis. The nomogram was formulated based on the results of multivariate analysis and by using the package of regression modeling strategies (rms) (https://cran.r-project.org/web/packages/rms/index.html) in the R language (https://www.r-project.org) (version 3.5.2).

## SUPPLEMENTARY MATERIAL

Supplementary Figure 1

Supplementary Table 1
