# Supplementary material for: Association of RAGE gene multiple variants with the risk for COPD and asthma in northern Han Chinese
Source: Aging (Albany NY). 2019 May 29;11(10):3220–37. doi: 10.18632/aging.101975 (PMC6555453; doi:10.18632/aging.101975)
Supplement: Supplementary Figure 1 [file aging-11-101975-s001.doc]

Supplementary Table 1. The primers of five studied variants in the gene encoding the receptor for advanced glycation end products.

| **Variants** | **Primers** | **Sequences** |
| --- | --- | --- |
| rs1800625 | Forward | 5’-AAAACATGAGAAACCCCAGAAAA-3’ |
|  | Reverse | 5’-GCATCATGAAGGCAAGGC-3’ |
| rs1800624 | Forward | 5’-AAGTTCCAAACAGGTTTCTCTCC-3’ |
|  | Reverse | 5’-CAAAGTTGCATCAATAGGGTTCAG-3’ |
| rs2070600 | Forward | 5’-GCTTGGAAGGTCCTGTCTC-3’ |
|  | Reverse | 5’-TCCATTCCTGTTCATTGCCTG-3’ |
| rs184003 | Forward | 5’-GGATGTGAGTGACCTGGAGA-3’ |
|  | Reverse | 5’-CTGCCTTTCCCTCGTTAGC-3’ |
| rs2071288 | Forward | 5’-GAATGGTGAGTGGTGGTGG-3’ |
|  | Reverse | 5’-AGAGTTCCCAGCCCTGAT-3’ |
