# Supplementary material for: Association of RAGE gene multiple variants with the risk for COPD and asthma in northern Han Chinese
Source: Aging (Albany NY). 2019 May 29;11(10):3220–37. doi: 10.18632/aging.101975 (PMC6555453; doi:10.18632/aging.101975)
Supplement: Supplementary Table 1 [file aging-11-101975-s002.pdf]

SUPPLEMENTARY MATERIAL

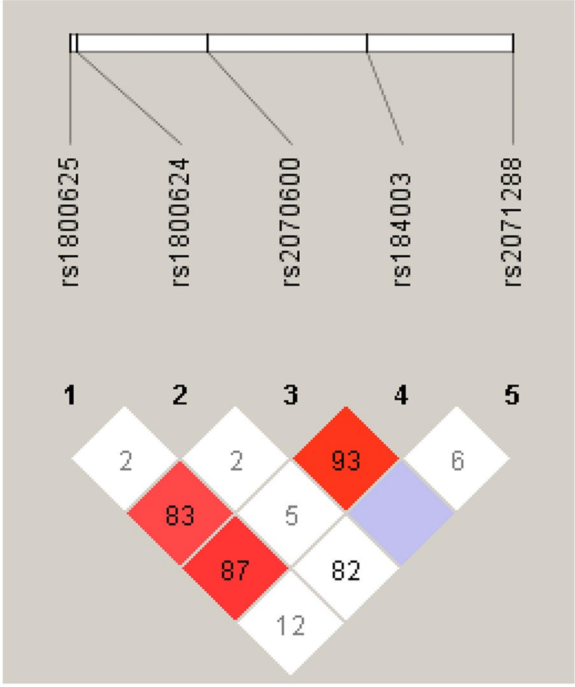

Supplementary Figure 1. Linkage disequilibrium graph of five studied variants in RAGE gene in northern Han Chinese. Pairwise linkage disequilibrium is expressed as D' (different colors) and r2 (numbers). Abbreviations: COPD, chronic obstructive pulmonary disease.
